# Two-Photon Laser Scanning Microscopy of the Transverse-Axial Tubule System in Ventricular Cardiomyocytes from Failing and Non-Failing Human Hearts

**DOI:** 10.4061/2009/802373

**Published:** 2010-03-07

**Authors:** Andreas Ohler, Jutta Weisser-Thomas, Valentino Piacentino, Steven R. Houser, Gordon F. Tomaselli, Brian O'Rourke

**Affiliations:** ^1^Department of Medicine, Institute of Molecular Cardiobiology, Johns Hopkins University, Baltimore, MD 21205, USA; ^2^Department of Cardiology and Pneumology, Georg-August-University Göttingen, 37075 Göttingen, Germany; ^3^Department of Physiology, Cardiovascular Research Center, Temple University School of Medicine, Philadelphia, PA 19140, USA; ^4^Department of Cardiology, University of Bonn, 53105 Bonn, Germany; ^5^Department of Medicine, University School of Medicine, Durham, NC 27705, USA

## Abstract

*Objective*. The transverse-axial tubule system (TATS) of cardiomyocytes allows a spatially coordinated conversion of electrical excitation into an intracellular Ca^2+^ signal and consequently contraction. Previous reports have indicated alterations of structure and/or volume of the TATS in cardiac hypertrophy and failure, suggesting a contribution to the impairment of excitation contraction coupling. To test whether structural alterations are present in human heart failure, the TATS was visualized in myocytes from failing and non-failing human hearts. *Methods and Results*. In freshly isolated myocytes, the plasmalemmal membranes were labeled with Di-8-ANEPPS and imaged using two-photon excitation at 780 nm. Optical sections were taken every 300 nm through the cells. After deconvolution, the TATS was determined within the 3D data sets, revealing no significant difference in normalized surface area or volume. To rule out possible inhomogeneity in the arrangement of the TATS, Euclidian distance maps were plotted for every section, allowing to measure the closest distance between any cytosolic and any membrane point. There was a trend towards greater spacing in cells from failing hearts, without statistical significance. *Conclusion*. Only small changes, but no significant changes in the geometrical dimensions of the TATS were observed in cardiomyocytes from failing compared to non-failing human myocardium.

## 1. Introduction

Regular cardiac function is dependent on coordinated contraction of all myocytes forming the heart. Since intercellular coordination is achieved by propagated electrical excitation and myofilament activation is calcium-dependent, contraction of the individual myocyte critically depends on a homogenous transduction of the action potential into an intracellular calcium signal. This first step of cardiac excitation contraction coupling (ECC) is mediated by voltage-dependent calcium channels embedded in the sarcolemma. Following excitation, influx of calcium ions triggers a sudden calcium release from the sarcoplasmic reticulum (SR), and the steep increase in cytosolic calcium concentration leads to activation of the myofilaments [1]. To ensure fast propagation of excitation and penetration of the calcium trigger signal to the cell interior, the sarcolemma contains deep invaginations into the cardiomyocyte, known as the transverse-axial tubule system (TATS). The distinct geometrical arrangement of the cell membrane also provides the important spatial relationship of key proteins of ECC, for example, positioning of the L-type Ca-channels approximately 12 nm opposite of SR calcium release channels [2].

A cellular hallmark of heart failure is the impairment of ECC. With a diminished calcium transient, consequent calcium-dependent myofilament activation is diminished, too. In human heart failure and animal models of chronic heart failure, significant alterations of calcium cycling proteins have been described, such as reduced expression and function of the SR Calcium ATPase and SR calcium release channels or increased expression of the sodium calcium exchanger [3, 4]. In addition, previous reports have indicated that both structure [5, 6] and volume [7, 8] of the TATS may be altered in cardiac hypertrophy and failure, suggesting a decreased efficiency of coupling between calcium entry and SR calcium release. To test whether such structural alterations are present in human heart failure, the TATS was visualized in cardiomyocytes isolated from failing and non-failing human hearts.

## 2. Materials and Methods

### 2.1. Cell Isolation

Human cardiomyocytes were enzymatically isolated from six human hearts (clinical data shown in Table 1), as previously reported [9, 10]. Briefly, four hearts were provided by patients who underwent heart transplantation for terminal heart failure in dilative cardiomyopathy (DCM), familial hypertrophic cardiomyopathy (FHCM), and ischemic cardiomyopathy (ICM). Two hearts were from healthy donors, in cases which the heart could not be transplanted for technical reasons. Non-failing hearts were obtained from Temple University's procurement organization, the Gift of Life. Informed consent to use organs for research was obtained from the patients prior to heart transplantation or for non-failing hearts from a donor family member at the time of death. All heart tissue specimens and data were deidentified to comply with HIPAA (Health Insurance Portability and Accountability Act) standards. All study related activities were conducted according to U.S. and international standards of Good Clinical Practice (Food and Drug Administration Title 21, part 312) and International Conference on Harmonization Guidelines. The investigation was carried out in accordance to the principles outlined in the Declaration of Helsinki of the World Medical Association and its amendments [11].

In each heart, a section of free left-ventricular wall was cut out and a branch of a coronary artery was cannulated for perfusion, on a customary Langendorff perfusion setup. After perfusion with of nominally calcium-free Krebs-Henseleit solution (KCl 5.4, lactic acid 1, MgSO_4_ 1.2, NaCl 130, NaH_2_PO_4_ 1.2, NaHCO_3_ 25, pyruvate 2, glucose 12.5; all mM; pH 7.4 adjusted with NaOH), the tissue was perfused with an enzyme solution containing 1 mg/ml Collagenase II, activated by 50 *μ*M calcium. On completion of the enzyme treatment, the tissue was washed in calcium-free solution, cut into chunks, and the isolated cells were harvested. The cells were stored in a solution containing a high potassium concentration (KCl 25, potassium glutamate 120, Hepes 10, EGTA 0.1, Glucose 10, MgCl_2_ 1; all mM; pH 7.25 adjusted with KOH) at 4°C and were used within 12 hours after cell isolation. In order to visualize the plasmalemmal membrane including the TATS, the cells were loaded with the fluorescence probe Di-8-ANEPPS (10 *μ*M, Molecular Probes) for 20 min in high potassium solution. The experiments were conducted at room temperature.

### 2.2. Imaging

**Figure 1:**
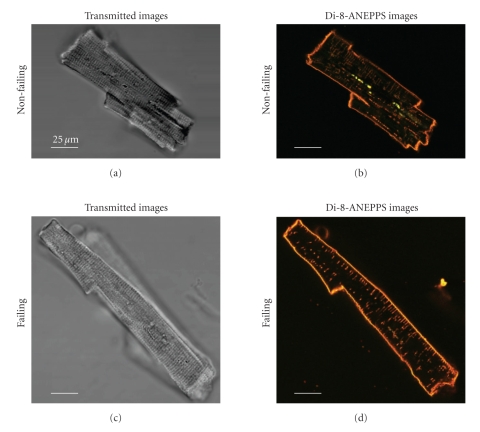
Optical Cross Sections of Human Cardiomyocytes from a Healthy Donor‘s Heart (a), (b) and an End-Stage Failing Heart (c), (d). Transmitted light images (a), (c) were recorded along with Di-8-ANEPPS fluorescence emission staining the plasma-lemmal membrane including the TATS (b), (d). These low power images were taken with a 60× lens without optical zoom. Autofluorescent “beads” were often observed in the perinuclear region (notable as greenish spots in the image shown in panel (b), showing a broad emission spectrum, even in the absence of Di-8-ANEPPS loading. These areas were excluded from the TATS analysis.

Immediately prior to the experiment, cells were mounted on a stage of an upright fluorescence microscope (E600FN, Nikon) on a two-photon laser scanning microscopy system (MRC-1024MP, Bio-Rad). Two-photon excitation of fluorescence was achieved by a tuneable Ti:Sapphire mode-locked laser (Tsunami, Spectra-Physics), fed by a 10 W a diode-pumped solid state laser (MilleniaX, Spectra-Physics), allowing laser emission between 700 nm and 980 nm. Excitation at 780 nm was found to be sufficient to excite Di-8-ANEPPS. Fluorescence emissions were collected in a two-channel external detector placed between the scan head and the microscope. The imaging system included software for data acquisition (Lasersharp, Bio-Rad) on a personal computer which was later analyzed using ImageJ (Wayne Rasband, National Institutes of Health). Transmitted light images were used to determine cell dimensions and sarcomere length. In every cell, a brightness profile along the longitudinal axis of the cell was analysed across 6 to 9 sarcomeres, and individual sarcomere length was calculated from distance and number of sarcomeres analysed. In order to visualize the entire TATS of individual cells, image series were obtained by focusing through the cells in increments of 300 nm with a motorized focus controller. Optically zooming into regions of interest increased the calculated pixel-resolution to 109.6 nm per pixel. The point spread function (PSF) of the optical system was determined by imaging fluorescent beads (Fluospheres, Molecular Probes) using the same settings. The full-width half-max of the PSF yielded values for the resolution of the optical system of ~300 nm in the *x* and *y* axes and ~700 nm in the *z*-axis. The PSF was then used in a kernel to deconvolve the images with a Weiner deconvolution algorithm on a computer workstation (Indigo 2, Silicon Graphics). Myocytes were randomly selected exclusively using transmitted light with exclusion of cells that appeared visibly damaged (i.e., not rod shaped) or did not show crisp striations. Only cells remaining mechanically quiescent during observation were used. Emitted fluorescence in the range of 580–630 nm was recorded along with transmitted light images (Figure 1). For the detailed analysis of the TATS, only deconvoluted images were used.

**Figure 2:**
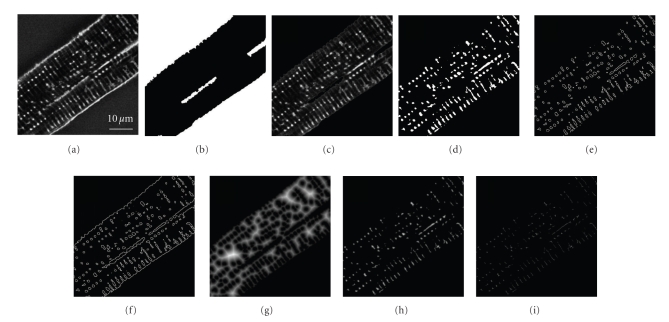
Image Processing for the Analysis of Cellular Volumes, Surface Areas, and Euclidian Distance Maps. From the deconvoluted images (a), binary masks (b) of the cell borders were drawn. The interior of the myocyte (c) was thresholded (d), with bright areas representing the TATS. The images were transferred into outline maps with or without cell borders, to estimate the surface area of the TATS (e) and the total membrane area of the cardiomyocyte (f). Furthermore, thresholded images were 2-fold inflated and transferred into Euclidian distance maps to determine the distance from any pixel representing cytosol to its next neighboring membrane (g), and respectively, from any pixel representing TATS to the next neighboring cytosol (h). Finally, the Euclidian distance maps of the TATS were reduced to their 3D-reconstructed skeleton in order to analyze regional object diameters (i). All Distance maps (g)–(i) are shown here brightened up from 4- to 32-fold for improved illustration.

For every section, cell borders were drawn to differentiate the peripheral sarcolemma from the interior of the cell. Thresholding these images led to binary masks of the objects inside the cell, from which area and perimeter of the objects could be calculated (Figure 2). The slices were summed to determine volume and surface area of the cell and the thresholded objects, representing the TATS. In order to analyze distances between cytosol and neighboring membranes or TATS diameters, the thresholded images were transferred into Euclidian distance maps (Figures 2 and 5).

As expected using this method, the results would be highly dependent on the choice of threshold. In these 8-bit grayscale images, one unit change in threshold would have resulted in a change of about 0.6% in TATS volume fraction. To avoid such subjective threshold selection errors, threshold values were calculated for every cell according to its pixel brightness distribution using the “Isodata method” [12]. Assuming that TATS and unstained cytosol would have two different distributions of intensity, we started from an arbitrary threshold and then the threshold value was iteratively replaced by the mean value of the distribution's averages above and below the given temporary threshold. Thereby, the final threshold value could be determined after 5 to 15 iterations.

### 2.3. Data Analysis

Pooled data are presented as mean ± SEM, and the number of cells or experiments is shown as “*n*”. Statistical comparison was evaluated by Student's *t*-test for unpaired samples, with a value of *P* < .05 considered to be significant.

## 3. Results

**Figure 3:**
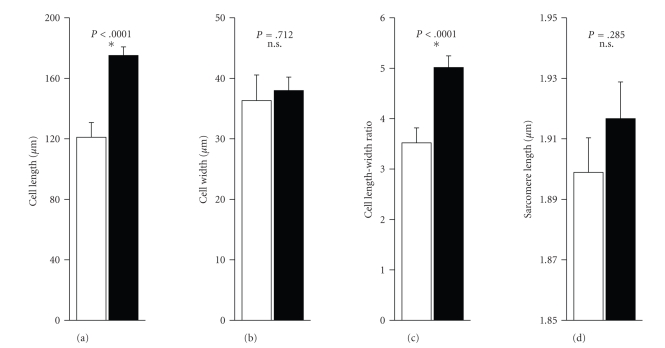
Geometrical Dimensions of Human Ventricular Myocytes from Failing and Non-Failing Hearts. Cell length and width were measured from transmitted light images of each cell taken with a 60× lens at 1- to 2-fold zoom (a,b). Cell length-width ratio was calculated from these data (c). Sarcomere length was measured from 6–9 sarcomeres in the transmitted light images (d). Open bars show the dimensions for myocytes from non-failing hearts (11 cells, 2 hearts), closed bars represent cells from failing human hearts (37 cells, 4 hearts). Calculated p-values as given, stars symbolize statistical significance.

Since cells from failing and non-failing hearts are known to show geometrical differences, low power transmitted light images of the cells were taken first to determine overall dimensions. Figure 1 shows typical transmitted light images, Di-8-ANEPPS fluorescence emission images of a cell from a healthy donor's heart, and an end-stage failing heart, respectively. Cell length was measured as the longest end-to-end distance along the longitudinal axis of the cell. Cell width was defined as the longest distance measured perpendicular to the longitudinal axis of the cell. Myocytes from failing human hearts were significantly longer, while cell width was not different (Figures 3(a)–3(c)). Mean sarcomere length measured from these transmitted light images showed no significant difference between cells from non-failing and failing hearts (1.899 ± 0.011 *μ*m versus 1.917 ± 0.012 *μ*m; Figure 3(d)).

**Figure 4:**
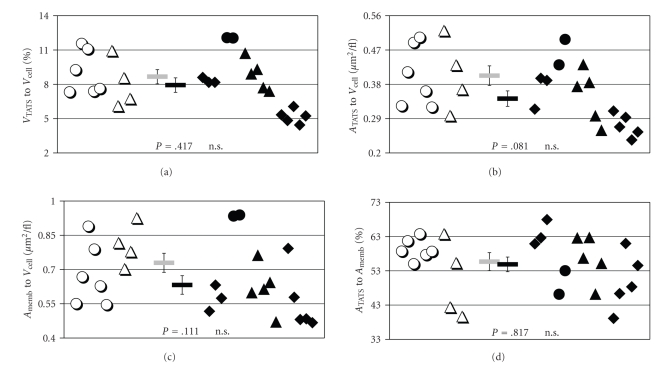
Volume and Surface Dimensions of the Transverse-Axial Tubule System. TATS volume (“V_TATS_”, (a)), TATS surface area (“A_TATS_”, (b)) and total cell membrane area (“A_Memb_”, (c)) are shown to be normalized to cell volume (“V_Cell_”). Also, TATS surface area is shown as the proportion to total cell membrane area (d). The results are given for all myocytes analyzed from non-failing (open symbols, *n* = 10, 2 hearts) and failing hearts (closed symbols, *n* = 15, 4 hearts). Different symbols denote different cell batches. Bar symbols in the middle of each chart indicate the mean values ± SEM. Calculated *P*-values as given.

In order to allow sufficient three dimensional resolution of the TATS, high power images (60× lens, 4-fold zoom) were taken in optical sections of 300 nm through the thickness of the cells. With these settings, the calculated pixel resolution was 109.6 nm, however, only a section of the cell, usually one third to one half, could be visualized. To allow a comparison between the groups, the results are expressed as ratios of cellular volume, TATS volume, or total membrane surface area (Table 2, Figure 4). 

Normalized for cell volume in a first comparison, no significant difference in volume or surface area of the TATS (Figures 4(a) and 4(b)) was observed between the myocytes from failing and non-failing hearts. These results are notable since surface and volume of intracellular structures are mathematically connected. It was already suggesting that with similar standard error of the mean, the geometrical structure of the TATS could not be fundamentally different between the groups either. However, there was a trend to smaller TATS surface area and total cell membrane surface area per cell volume in myocytes from failing hearts (Figures 4(b) and 4(c)). Consequently, no significant difference could be found in the ratios of total cell membrane surface to cell volume or TATS membrane surface to total cell membrane surface (Figures 4(c) and 4(d)).

**Figure 5:**
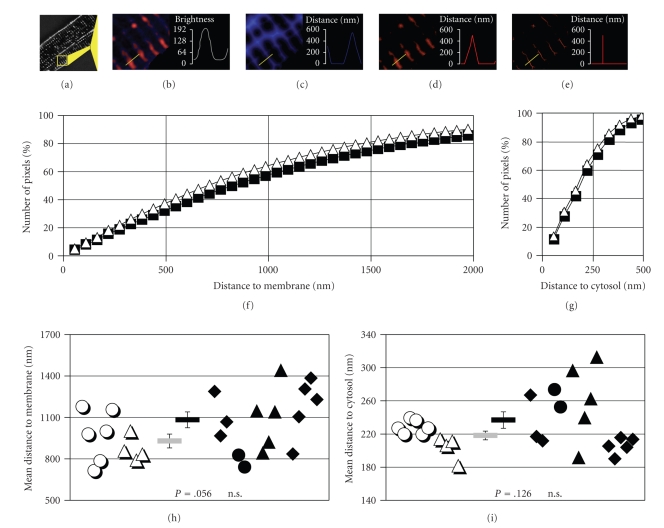
Distance Distribution of the Transverse-Axial Tubule System. Every image of a cell ((a), scaled up and colored in (b)) was transferred into Euclidian distance maps. The distance from any pixel representing cytosol (blue) to its next neighboring membrane (red) within the image was analyzed and then plotted as a brightness value in the distance map (c). The Euclidian distance maps, of the TATS were developed within 3D recontructions of the TATS (d) and reduced to their 3D-reconstructed skeleton in order to analyze regional object diameters (e). From cytosolic distance maps the average of 22 × 10^6^ pixels (non-failing) versus 50 × 10^6^ pixels (failing) was plotted as normalized number of pixels against distance to membrane (f). From TATS distance maps, the average of 2.1 × 10^6^ pixels (non-failing) versus 4.4 × 10^6^ pixels (failing) was plotted as normalized number of pixels against distance to the next neighboring cytosolic pixel (g). Weighted means of the measurements resolve the mean distance between cytosol and membrane (h), and respectively, the mean object diameter (i). Open symbols represent measurements from non-failing hearts (2 hearts, 10 cells, 467 slices), closed symbols from failing hearts (4 hearts, 15 cells, 872 slices). Different symbols denote different cell batches. Bar symbols in the middle of chart (h) and (i) indicate the mean values ± SEM. Calculated *P*-values as given. Exemplary distance maps (b)–(d) are shown brightened up for improved illustration.

However, the lack of significant differences in the geometrical analysis does not exclude inhomogeneity in the arrangement of the TATS in cells from failing hearts, as previously described in a canine model of heart-failure [7, 8]. Therefore, every optical section of the myocytes was transformed into Euclidian distance maps (Figures 5(a)–5(e)). From these maps, a trend towards greater spacing in myocytes from failing hearts was observed, but did not reach statistical significance (930 ± 50 nm versus 1083 ± 57 nm, Figure 5(h)). Combining the distances of all “cytosolic” pixels, roughly 60% of the pixels were found within a distance of half a sarcomere and over 80% within a distance of one sarcomere (Figure 5(f)). Only for distances above 3000 nm, that is, less than 5% of the cytosolic pixel distribution, significant differences between cells from non-failing and failing hearts could be observed. Likewise, the cumulative distances of the TATS (Figure 5(g)) did not show significant differences up to 550 nm, representing more than 97% of the TATS pixel distribution. Summarizing the distances from the skeletonized 3D reconstructions only, the mean radius of the transverse-axial tubule system was found to be 210 ± 6 nm versus 228 ± 10 nm, again statistically without significant difference (Figure 5(i)).

## 4. Discussion

The transverse-axial tubule system was discovered by Lindner [13] in dog myocardium. Numerous studies using electron microscopy (EM) followed, observing the TATS in different mammal species, including mouse, guinea-pig, rat, rabbit, cat, and human myocardium [14]. However, EM quantitative data on the TATS are difficult to obtain, since high resolution imaging implies observation of small myocyte volumes, and electron beam penetration into the cells is limited. Moreover, the specimens require special preparation that may distort the structure of the cellular membranes and the TATS.

On the other hand, conventional microscopy using visible wavelengths cannot resolve cell membranes with a diameter of 7 nm, nor the t-tubule of about 50–150 nm in diameter [15]. In the present study, we visualized the TATS using the dye Di-8-ANEPPS. Recently, the emission of such voltage-sensitive indicators has been reconfirmed and quantified for two-photon excitation [16]. The advantage of two-photon fluorescence excitation is that it is limited to the focal plane and does not require correction for out-of-focus fluorescence, improving signal-noise ratio. We further enhanced resolution of the TATS by deconvolution of the images to correct for the blurring inherent in the optical system.

However, the images we analyzed still reflect the emission of excited fluorescence and do not directly show the cellular membranes. Thus, setting the threshold of the images is crucially important: increasing the threshold by one unit in our images would have decreased the calculated TATS volume fraction by about 0.6%, that is, roughly 10% of the final result. This value is comparable with values re-calculated from published images of other groups [17, 18]. To ensure independent thresholding of the images, we applied the “Isodata method” [12] (see Materials and Methods).

### 4.1. The Transverse-Axial Tubule System in Non-Failing Myocardium

Since this is the first detailed quantitative study on the transverse-axial tubule system of human ventricular cardiomyocytes, comparable numbers are rare or are only given for other species. In cells from non-failing human myocardium, we observed the TATS to occupy 8.7 ± 0.6% of the cellular volume, spanning from 6.1% to 11.6%. Louch et al. [19] have measured a TATS volume fraction (V_TATS_/V_Cell_) of up to 26% from three slices each of 14 myocytes from failing human heart. The considerable difference is possibly due to the use of undeconvoluted images, a different method of thresholding, and maybe partially out of focus fluorescence. Lyon et al. [20] calculated a t-tubule density of 68% in single slices from 8 myocytes from non-failing human heart as the ratio between TATS and whole cell fluorescence without thresholding. Therefore, t-tubule density cannot be compared directly to our measurements. The method elegantly ensures independence from thresholding, but could be susceptible to inhomogeneous loading of the dye, fluorescence gain, and overlay in fluorescence. 

In isolated myocytes from non-failing canine myocardium, TATS volume fractions of 10% [8] and 11.5% [7] respectively were found. For other mammalian cardiomyocytes, quite non-uniform results have been published (V_TATS_/V_Cell_ guinea-pig: 7.7% [21], 2.5–3.2% [15, 22]; rabbit: 3.2–3.6% [23]; mouse: 0.81% [24], 3.3% [25]). Likewise, in rat myocardium, earlier quantitative EM studies found V_TATS_/V_Cell_ ratios of only about 1% [26, 27], while using two-photon laser excitation of Di-8-ANEPPS, 3.6% [17] was reported. For all of these quantitative EM studies, the authors recognized the limitations imposed by the considerable influence of specimen preparation. Data on TATS surface density (V_TATS_/V_Cell_ guinea-pig: 0.38–0.48; [15, 22] rabbit: 0.16–0.24 [27], 2.46 [23]; rat: 0.08–0.19 [27], 0.34 [26], 0.40 [17]; mouse: 0.50 [24]; all *μ*m²/*μ*m^3^) show similar diversity, and reports on the proportion of TATS surface area to myocyte total membrane (V_TATS_/V_Memb_: guinea-pig: 53% [15]; rabbit: 33–41% [27]; rat: 38%–65% [17, 26, 27]) are rare. However, our measurements for non-failing heart are in good agreement with the numbers mentioned above (V_TATS_/V_Cell_: 0.40 ± 0.03 *μ*m²/*μ*m^3^; V_TATS_/V_Memb_ 55.7 ± 2.6%).

### 4.2. The Transverse-Axial Tubule System in Failing Myocardium

The significance of the transverse-axial tubule system for functional electro-mechanical coupling has been characterized in detail. Key proteins of ECC and of membrane potential control, like L-Type Ca^2+^ channels, Na^+^/Ca^2+^ exchanger, *α*_1_-Na^+^/K^+^ ATPase, Na^+^, and certain K^+^ channels, G_s_-protein, adenylate cyclase or pH-regulating proteins, have been reported to be concentrated in the membranes of the TATS [28]. Accordingly, disruption or degradation of the TATS by chemical treatment (“detubulation”) or cell culture was linked to critical impairment of ECC [29–31]. Alterations in the expression and arrangement of cytoskeletal proteins have also been reported in both human and animal models of heart failure. From cytoskeleton data and cell capacitance measurements [32], it was suggested that the TATS could be altered as well, with the functional consequences mentioned above. On the other hand, observation of T-index and calcium transients in myocytes from human failing heart did not considerably differ from freshly isolated pig cardiomyocytes (since non-failing human tissue was not available) and raised doubt about a pronounced loss of T-tubules in failing human heart [19]. Lyon et al. [20] describe a significant reduction of Di-8-ANEPPS fluorescence in myocytes from failing compared to non-failing hearts along with slowing of contraction and relaxation times, but unfortunately the study could not measure the dimensions of the TATS. 

In animal models of heart failure, Kamp and coworkers visualized the TATS in a canine tachycardia-induced model of heart failure, analyzing volume fraction only [7, 8]. Interestingly, the observed significant reduction of TATS volume fraction (10% versus 5% [8]; 11.5% versus 8.3% [7]) in cells from failing heart was mainly due to a loss of TATS structure at the extremities of the cells, rather than a homogenous reduction. Despite small trends, significant changes in fractional volume, surface density, and the proportion of TATS surface area relative to the total cellular membrane could not be detected in our experiments.

### 4.3. Spatial Homogeneity of the Transverse-Axial Tubule System

The main purpose of the transverse-axial tubule system is presumably to allow fast propagation of the action potential into the interior of the myocyte. The distance of any point in the cell interior to the next neighboring membrane, as expressed in our Euclidian distance maps, should therefore be an indicator of the homogeneity of membrane distribution. There was a trend towards greater spacing in myocytes which failed to be statistically significant up to 3000 nm. Since we found sarcomere length to be slightly longer in myocytes from failing human heart, a slightly greater spacing would have been expected. The mean diameter of the TATS, measured around 450 nm, was found to be considerably larger compared to animal or EM studies.

### 4.4. Limitations of the Study

In summary, we found no convincing evidence for significant alterations in the dimensions of the TATS in isolated ventricular cardiomyocytes from failing compared to non-failing human myocardium. As for all comparative studies in human ventricular myocardium and limited sample size, there remains some doubt as to whether the cells from non-failing hearts are completely unchanged, and whether different pathologies of heart failure would demonstrate similar findings. Another potential limitation is that the myocytes most dramatically affected in the failing heart may be more sensitive to damage or death during the cell isolation procedure, introducing bias into the selection process. Thus, future studies could employ the advantages of multiphoton laser scanning microscopy to visualize the transverse-axial tubule system in intact myocardium, rather than isolated myocytes.

## Figures and Tables

**Table 1:** *Clinical Characteristics of the Patient Population.* Four hearts (Pat #1–4) were provided by patients who underwent heart transplantation for terminal heart failure in dilative cardiomyopathy (DCM), familial hypertrophic cardiomyopathy (FHCM), or ischemic cardiomyopathy (ICM). Two hearts (Pat #5-6) were from healthy donors.

	Gender	Age	Patient	Patient	BMI	Disease	EF	Heart	Medication	Number of
			weight	height				weight		cells used
	[f/m]	[years]	[kg]	[cm]	[kg/m²]		[%]	[g]		
Pat. no.1	f	59	62	170	21	DCM	—	433	Milrinone, ACEI, Ang II Antag, *α*Blocker, Ca-Blocker, Digitalis, Diuretics	3
Pat. no.2	f	27	57	152	25	FHCP	10%	369	Milrinone, ACEI, *β*-Blocker, Digitalis, Diuretics	2
Pat. no.3	f	31	76	163	29	DCM	13%	486	Milrinone, ACEI, Nitrate, Digitalis, Diuretics	5
Pat. no.4	m	62	67	178	21	ICM	18%	417	Milrinone, ACEI, Nitrate, Digitalis, Diuretics	5
Pat. no.5	f	57	60	152	26	donor	60%	360	Dobutamine	6
Pat. no.6	f	16	52	175	17	donor	53%	373	Milrinone	4

**Table 2:** *Volume and Surface Area Dimensions of the Transverse-Axial Tubule System.* Fractions were calculated individually for all cells before averaging. Regions containing the cell's nucleus or autofluorescent beads within the images were excluded before quantitative analysis.

	Unit	Non-failing	Failing	*P*
V_TATS_/V_Cell_*	[%]	8.67 ± 0.62	7.93 ± 0.64	*P* = .417
A_TATS_/V_Cell_	[*μ*m²/*μ*m^3^]	0.403 ± 0.026	0.343 ± 0.020	*P* = .081
A_Cell_/A_Cell_	[*μ*m²/*μ*m^3^]	0.327 ± 0.033	0.290 ± 0.028	*P* = .404
A_Memb_/V_Cell_	[*μ*m²/*μ*m^3^]	0.729 ± 0.042	0.632 ± 0.041	*P* = .111
A_TATS_/V_TATS_	[*μ*m²/*μ*m^3^]	4.682 ± 0.123	4.476 ± 0.197	*P* = .386
A_TATS_/A_Memb_	[%]	55.68 ± 2.63	54.89 ± 2.12	*P* = .817

V_Cell_	Apparent cellular volume, that is, volume surrounded by the exterior cellular membrane			
V_TATS_	Intracellular volume occupied by the TATS			
A_TATS_	Surface area of the TATS			
A_Cell_	Surface of the exterior cellular membrane			
A_Memb_	Total membrane surface area, that is, A_Cell_ + A_TATS_			

*in previous studies *V*_TATS_/*V*_Cell_ ratio is referred to as fractional volume, TATS volume fraction, or *t* index.
